# Development of 2′-O-Methyl and LNA Antisense Oligonucleotides for *SMN2* Splicing Correction in SMA Cells

**DOI:** 10.3390/biomedicines11113071

**Published:** 2023-11-16

**Authors:** Marianna Maretina, Arina Il’ina, Anna Egorova, Andrey Glotov, Anton Kiselev

**Affiliations:** 1 Department of Genomic Medicine Named after V.S. Baranov, D.O. Ott Research Institute of Obstetrics, Gynecology and Reproductology, Mendeleevskaya Line 3, 199034 Saint Petersburg, Russia; marianna0204@gmail.com (M.M.); egorova_anna@yahoo.com (A.E.); anglotov@mail.ru (A.G.); 2 Faculty of Biology, Saint Petersburg State University, Universitetskaya Embankment 7–9, 199034 Saint Petersburg, Russia; arina-ilina-23@yandex.ru

**Keywords:** spinal muscular atrophy, *SMN1* gene, *SMN2* gene, nuclear gems, antisense oligonucleotides, splicing correction, 2′-O-methyl modification, LNA

## Abstract

Spinal muscular atrophy (SMA) is a devastating neurodegenerative disease caused by mutations in the *SMN1* gene. Existing therapies demonstrate positive results on SMA patients but still might be ameliorated in efficacy and price. In the presented study we designed antisense oligonucleotides (AONs), targeting intronic splicing silencer sites, some were modified with 2′-O-methyl, others with LNA. The AONs have been extensively tested in different concentrations, both individually and combined, in order to effectively target the ISS-N1 and A+100G splicing silencer regions in intron 7 of the *SMN2* gene. By treating SMA-cultured fibroblasts with certain AONs, we discovered a remarkable increase in the levels of full-length *SMN* transcripts and the number of nuclear gems. This increase was observed to be dose-dependent and reached levels comparable to those found in healthy cells. When added to cells together, most of the tested molecules showed a remarkable synergistic effect in correcting splicing. Through our research, we have discovered that the impact of oligonucleotides is greatly influenced by their length, sequence, and pattern of modification.

## 1. Introduction

Spinal muscular atrophy (SMA) is a severe neuromuscular disorder with an incidence of 1:6000–10,000 births and a carrier frequency of 1:40–1:60 [1]. It is one of the prominent genetic causes of infant mortality and is subdivided into five clinical types (0–IV) depending on the time of symptom initiation and their severity [2]. Progressive muscle weakness is the main symptom that develops as a result of degeneration of the motor neurons of the spinal cord. In most severe cases like 0 or I types of SMA that develop prenatally or in the first months of life additional symptoms like heart defects have been described [3]. SMA type I is the most frequent subtype of SMA that is characterized by onset before 6 months of life, no ability to sit unsupported, and not more than 2 years lifespan in the absence of treatment. SMA type II develops between 7 and 18 months of age. Patients achieve the ability to sit unaided but they are not able to walk independently. SMA type III (Kugelberg–Welander disease) includes a clinically heterogeneous group of patients, joined by their ability to stand and walk. Depending on the time of weakness onset (before 3 years of age for SMA IIIa subtype or after this age for SMA IIIb) the time of losing the ability to walk. Meanwhile, SMA type IV—the rarest form—is a mild adult form characterized by normal life expectancy. The range of SMA phenotypes between the most severe and mild forms is actually a continuous spectrum that is conditionally subdivided into common subtypes based on age of onset and achieved motor milestones [4].

SMA is caused by survival motor neuron 1 (*SMN1*) homozygous mutations [5]. *SMN2* is a nearly identical gene that differs from *SMN1* by 14 single nucleotide changes and one insertion which produces the same SMN protein but in lesser quantity [6]. The principal difference is C-to-T substitution in exon 7 of *SMN2* which leads to disruption of splicing and production of exon 7-lacking transcripts in approximately 90% of cases [7]. In SMA patients the remaining 10% of *SMN2* transcripts are not enough to compensate for the lack of *SMN1* transcripts. The *SMN2* gene is the main disease modifier and the principal target for therapy approaches aimed at stimulating SMN production [8]. Spinal muscular atrophy severity can also be influenced by various modifiers, including epigenetic modifications, different factors that interact with SMN function and promote motor neuron survival, as well as transcriptional and splicing factors that can influence the expression of *SMN2* [9,10]. In the nucleus, SMN protein accumulates in structures called “gems” that are responsible for snRNP biogenesis and are detectable under a microscope as distinct dots [11]. Previously it was shown that full-length *SMN* (*FL-SMN*) transcript level and gems number inversely correlate with SMA severity [12,13]. We demonstrated that these values may be used as perspective biomarkers of SMA [14,15].

To date, there are few FDA-approved therapeutic agents for SMA including the antisense oligonucleotide (AON) drug nusinersen (Spinraza; Biogen), viral-vector-based gene therapy onasemnogene abeparvovec (Zolgensma; Novartis) and small molecule splicing modifier risdiplam (Evrysdi; Roche). Approved in 2016, Nusinersen stands as the pioneering treatment for SMA, solidifying antisense therapy as one of the utmost successful methods for combating spinal muscular atrophy. Several highly effective AONs have been described and many more are still being researched. Existing drugs like Spinraza are not without limitations, particularly the need for repetitive intrathecal injections [16]. Moreover, there are concerns related to hepatotoxicity and affordability with drugs such as Zolgensma, as they are limited to being accessible only to patients under the age of 2 [17,18]. Another factor to consider is that Evrysdi may have off-target splicing regulation and potential side effects [19]. Furthermore, the combination of various drugs that induce SMN is believed to enhance therapeutic outcomes [20]. Nevertheless, this approach is highly debatable [21].

**Figure 1:**
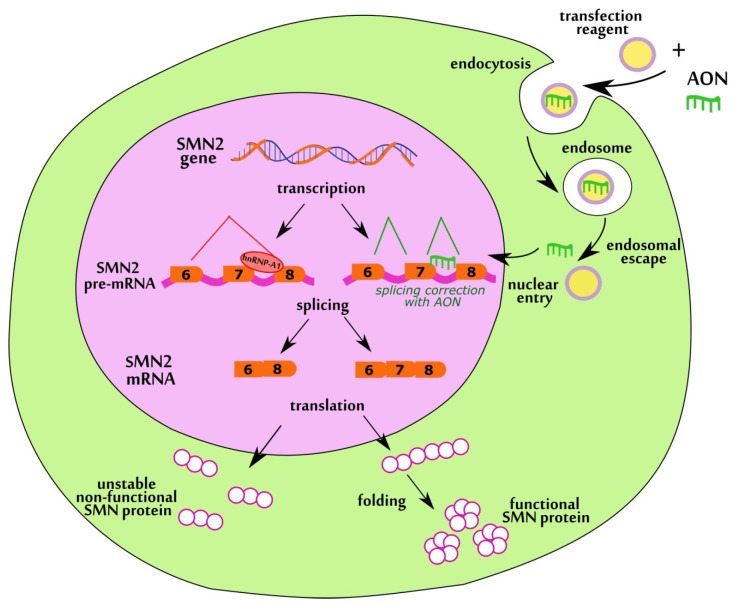
Mechanism of action of AONs aimed at correction of *SMN2* splicing.

AONs are short single-stranded nucleic acid molecules that can effectively modify the splicing of pre-mRNA through complementary base pairing (Figure 1). Their maximum effect can be achieved by designing optimal sequence, length, and modification that can be changed in a wide range. Most of the AONs for *SMN2* splicing correction including nusinersen target the intronic splicing silencer N1 (ISS-N1) located immediately downstream of the 5′ splice site in intron 7 [22,23,24]. This element was found to be a key negative regulator of exon 7 splicing and its suppression with AONs restored exon 7 inclusion in *SMN2* transcript and increased SMN protein level [25]. Other inhibitory regions influencing exon 7 splicing have also been described, such as Element 1 in intron 6, ISS-N2 and ISS+100 in intron 7, 3′ splice site of exon 8, whose masking increased *FL-SMN* transcripts level [26,27,28,29]. In the study by Pao et al. simultaneous targeting of two nonadjacent splicing silencers resulted in the efficient restoration of *SMN* transcripts and protein level [30].

For efficient delivery in vitro and in vivo, stability in cells, and optimal manifestation of therapeutic properties AONs require additional chemical modifications. Different modifications can be made to oligonucleotides, enhancing their properties. Modifications of the phosphodiester backbone (phosphorothioate (PS), phosphorodiamidate morpholinos (PMOs)) enhance nuclease stability and pharmacokinetic properties, modifications of the sugar ring (2′-O-methyl (2′OMe), 2′-O-methoxyethyl (MOE), locked nucleic acid (LNA)) modulate binding affinity, nuclease stability and interactions with cellular proteins, modifications to the nucleobases influence base-pairing specificity [31]. AONs with these and other modifications demonstrated encouraging results in preclinical and clinical studies of antisense therapies for neurodegenerative and other disorders and 18 AONs were successfully approved for treatment [32,33].

Nusinersen is an 18 nucleotide (nt) long 2′-O-(2-methoxyethyl) (MOE) phosphorothioate-modified AON. It demonstrates satisfying efficacy in treating SMA but has a very high price. Promising results were also obtained with PMO, 2′OMe, and LNA oligonucleotides tested as potential SMA therapies [22,24,34]. Currently, significant efforts are being made to develop a better variant of AON by using different strategies e.g., nusinersen oligomerization, targeting of two or more ISS in intron 7 of *SMN2* gene, application of saRNA targeting *SMN2* promoter, using a delivery system to penetrate the blood-brain barrier, etc. [30,35].

In this study, we aimed to devise therapeutic oligonucleotides that will be efficacious, non-toxic, and at the same time easily synthesized and cost-effective. We designed several AONs with different lengths, complementary to different negative splicing regulatory elements (SREs), that negatively regulate *SMN2* exon 7 splicing, and with different chemical modifications (PS, 2′OMe and LNA) and tested them on SMA fibroblasts cultures.

## 2. Materials and Methods

The study was performed using large-scale research facilities #3076082 “Human Reproductive Health” in D.O. Ott Research Institute of Obstetrics, Gynecology and Reproductology. Primary fibroblast cell cultures were obtained from a skin biopsy of a patient with SMA type II and a healthy individual. Cell cultures were incubated in DMEM with L-glutamine and 4.5 g/L glucose (Biolot LLC, Saint-Petersburg, Russia), supplemented by 10% fetal bovine serum (FBS, Gibco, Grand Island, NY, USA) and antibiotic (Biolot LLC, Saint-Petersburg, Russia) (penicillin 100 U/mL, streptomycin 100 μg/mL), at 37 °C in 5% CO_2_ as described previously [36]. Informed consent was obtained from the participants of the study. SMA cell culture was tested for the presence of a deletion in the *SMN1* gene and for the number of *SMN2* gene copies as described previously [37]. Homozygous *SMN1* exon 7 deletion was confirmed and 3 copies of *SMN2* gene were revealed.

All AONs were synthesized by Syntol JSC (Moscow, Russia).

### 2.1. Transfection

SMA fibroblasts were seeded 24 h prior to transfection on a 24-well plate (for immunocytochemical analysis, in a 48-well plate with 9-mm-diameter gasses on the bottom of the wells) in DMEM containing L-glutamine and 10% FBS without adding antibiotic. The percent of seeded cells was calculated so that they achieve the density of ~50% confluency on the day of transfection. Transfections of fibroblasts were performed with RNA antisense oligonucleotides using the XtremeGENE transfection reagent (Roche, Mannheim, Germany) according to the manufacturer’s instructions. AONs previously reported by Singh and colleagues served in the experiments as controls: 3UP8 oligonucleotide in 2′-O-methyl modification restoring *SMN2* exon 7 splicing as a positive control and F8 oligonucleotide in 2′-O-methyl modification as a negative control [22]. A 10:1 ratio of XtremeGENE (μL) to RNA (μg) which was shown to be most effective in the previous study was used in the current research [14]. Each RNA+transfection reagent complex was added to cells in duplicate. Transfected cell cultures were incubated for 4 h at 37 °C in 5% CO_2_, after that the medium was changed to the full one (with L-glutamine, FBS, and antibiotic) and then cells were maintained for 48 h in a CO_2_ incubator. After that, the fibroblasts were taken off from the culture surface of the plate and then RNA was isolated as described in Section 2.3. All transfection experiments were repeated at least 2 times.

### 2.2. Analysis of Toxicity of RNA/Carrier Complexes

SMA fibroblasts were seeded on a 96-well plate and transfected the next day similar the same way as in a 24-well plate but in 5.7 times less volume. 4 h after the addition of complexes the medium was changed to full containing a 10% solution of Alamar Blue (BioSource, Camarillo, CA, USA). The plate with the cells was incubated in a CO_2_ incubator for 16 h. The fluorescence intensity was measured on a Wallac 1420D fluorimeter at wavelengths of 530/590 nm. The relative number of cells after the addition of complexes was calculated by the formula (F − Ff)/(Fb − Ff) × 100%, where Fb is the fluorescence intensity of the Alamar Blue dye in the absence of DNA/carrier complexes and Ff is the fluorescence intensity of the dye in the absence of cells.

### 2.3. RNA Isolation and cDNA Synthesis

Fibroblasts in a 24-well plate were washed twice with 200 µL of PBS and then removed from the culture surface by incubation in 200 µL of a Trypsin–Versen Mixture (1:3) (Biolot LLC, Saint-Petersburg, Russia) for 10 min at 37 °C. Then 300 µL of PBS was added to the wells, mixed, transferred to tubes, and centrifuged for 10 min at 2200 rpm (+4 °C). The supernatant was discarded, and 125 µL of TRIzol reagent (Invitrogen, Burlington, Ontario, Canada) was added to the residue and resuspended. After 5 min incubation at room temperature (RT), 25 µL of chlorophorm was added, the contents of the tube were mixed, incubated for 3 min, and then the tubes were centrifuged for 15 min at 12,000 rpm (+4 °C). The upper phase was transmitted to a new tube and then RNA was precipitated with 63 µL of isopropanol. The tubes were placed in a freezer at −70 °C for a night. The next day tubes were centrifuged at 10,000 rpm for 20 min (+4 °C) and the supernatant was discarded. The residue was washed with 125 μL of cold 70% ethanol and centrifuged for 3 min at 14,000 rpm (+4 °C). The supernatant was discarded and RNA precipitate was left to dry for 1 h at RT. The RNA pellet was dissolved with 20 μL of DEPC-treated H_2_O and then incubated for 40 min at RT.

About 500 ng of total RNA was reverse transcribed using the first strand cDNA synthesis kit with random primers (Sileks, Moscow, Russia) according to the manufacturer’s protocol.

### 2.4. Semiquantitative and Quantitative Fluorescence RT-PCR

1 µL of cDNA was added to the PCR mix that included 1 µL of 10× PCR buffer with MgCl2, 0.31 mM of each dNTP, 1 µM of forward and reverse primer, 5 U of Taq DNA polymerase (SibEnzyme, Novosibirsk, Russia). *SMN* F 5′-GTCCAGATTCTCTTGATGAT-3′, complementary to *SMN* exon 6, and *SMN* R 5′-CTATAACGCTTCACATTCCA-3′, complementary to *SMN* exon 8 were used for *FL-SMN* and exon 7-deleted (*Δ7-SMN*) transcripts amplification. When performing quantitative fluorescence PCR (QF-PCR) the forward primed was labeled with FAM dye. The amplification reaction was conducted at the following conditions: 94 °C for 4 min, n cycles of 94 °C for 45 s, 50 °C for 45 s, 72 °C for 45 s, and final synthesis at 72 °C for 8 min. The number of amplification cycles did not exceed 29. PCR with each cDNA sample was conducted at least 2 times.

PCR products obtained after semiquantitative RT-PCR were separated on 6% polyacrylamide gel, stained with a ethidium bromide solution (0.5 μg/mL), and photographed on a transilluminator in transmitted UV light (wavelength 380 nm). The luminescence intensity of the PCR products was assessed in ImageJ version 1.54d (NIH, Bethesda, MD, USA).

For analyzing the results of QF RT-PCR, 1 μL of the amplification product was added to 12 μL of formamide (MCLAB, San Francisco, CA, USA) and 0.25 μL of the molecular weight marker LIZ500 (ABI, Foster City, CA, USA), and then the fragments were separated in ABI 3130xl capillary electrophoresis instrument at 60 °C. The visualization of the results was carried out using GeneMapper software version 3.7 (ABI, Foster City, CA, USA).

### 2.5. Immunocytochemistry

Round 9-mm glasses with cells were taken out of 48-plate wells and placed to coverslips. Cells were washed with PBS and fixed with 4% paraformaldehyde; PBS at RT for 10 min. After that, the glasses were washed with PBS (2 times, 2 min incubation each time) and incubated in 0.1% Triton X-100; PBS at RT for 5 min. Then the glasses were washed by incubating in PBS for 5 min. To reduce the background fluorescence, the glasses were blocked in 1% BSA in PBS for 1 h at RT. After that, the gasses were stored with 5 µg/mL of SMN antibody dilution (Novus Biologicals, Littleton, CO, USA) and prepared in 1% BSA overnight at 4 °C. The next day the glasses were washed in PBS three times for 5 min each and then incubated with the secondary antibody dilution (R&D Systems, Minneapolis, MN, USA) 1:200 in 1% BSA for 1 h at RT in a dark environment. After washing with PBS for three times 5 min each the glasses were dried, and the mounting medium with DAPI was added (Vector laboratories, Burlingame, CA, USA). Then another glass was put atop the coverslip and sealed with nail polish. The analysis was performed on a fluorescent microscope (Leica DM 2500) (Leica Microsystems, Wetzlar, Germany) with 1000× magnification. The number of gems was estimated per 100 nuclei.

### 2.6. Statistical Methods

Diagrams were done using GraphPad PRISM 8.0.2 (GraphPadSoftware Inc., San Diego, CA, USA). The Mann–Whitney U-test was used for statistical comparisons.

## 3. Results

### 3.1. Design of Antisense RNA Oligonucleotides Complementary to Negative Splicing Elements of SMN2 Gene

**Figure 2:**
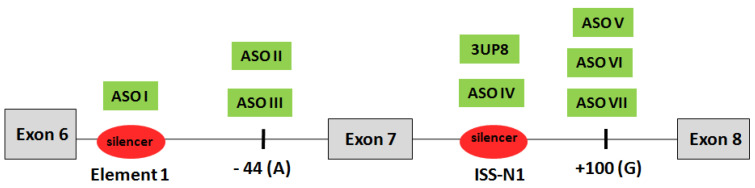
Scheme of AONs targets in *SMN2* pre-mRNA.

At the first step of our study, we designed RNA AONs with phosphorothioate and 2′-O-methyl modifications complementary to exon 7 negative splicing elements located in intron 6 and intron 7 of the *SMN2* gene (Table 1). We took 3UP8 as a positive control based on the study from Singh et al. who showed that this AON blocks ISS-N1 and restores *SMN2* exon 7 inclusion [22]. Also, we took the same RNA sequence as Nusinersen and made it with PS-2′OMe modified (ASO IV). Three AONs were tested for ISS+100 negative SRE: ASO V was designed by Pao et al. and two others were designed by us—14 nt-long ASO VI and 20 nt-long ASO VII (Figure 2).

ASO I with sequence taken from the study of Osman et al. targets Element 1 [38]. ASO II and III were designed for the first time to mask the −44 region described by Wu et al. as a HuR-dependent splicing silencer [39].

**Figure 3:**
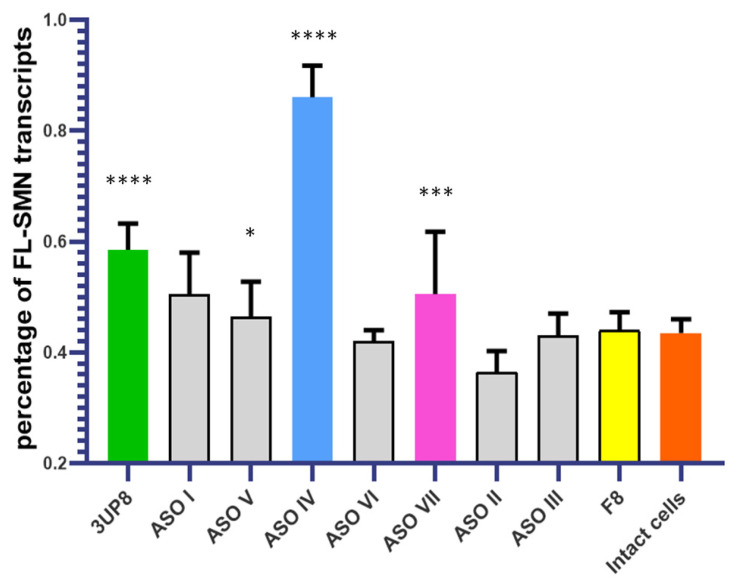
Percentage of full-length *SMN* transcripts in SMA fibroblast cell culture after delivery of AONs. * *p* < 0.05, *** *p* < 0.001, **** *p* < 0.0001. The comparison was performed relative to intact cells. Medians with interquartile range are given.

To assess the AONs effect we performed the transfections of SMA fibroblasts. Optimal RNA/carrier ratio and AON concentrations were chosen based on our previous results [14]. We delivered each in SMA fibroblasts each AON at a concentration of 200 nM and picked up ASO IV and ASO VII as oligonucleotides most efficiently restoring *SMN2* splicing (Figure 3). *SMN2* splicing correction efficacy was determined by means of the percentage of *FL-SMN* transcripts relative to the total sum of *FL-SMN* and *∆7-SMN* transcripts detected by semiquantitative and quantitative fluorescence RT-PCR [14].

ASO IV and ASO VII have been selected for further analysis and testing to be compared with AONs that have the same sequence but different chemical modifications. We have developed enhanced versions of these AONs by incorporating four LNA modifications. We added two LNA nucleotides at the 5’ end and another two LNA nucleotides at the 3’ end. Additionally, we created nLNA-modified versions by alternating common ribonucleotides with LNA nucleotides (Table 1). In addition, we made similar modifications to our positive control 3UP8 and negative control F8.

### 3.2. Evaluation of the AONs Efficiency at Different Concentrations

**Figure 4:**
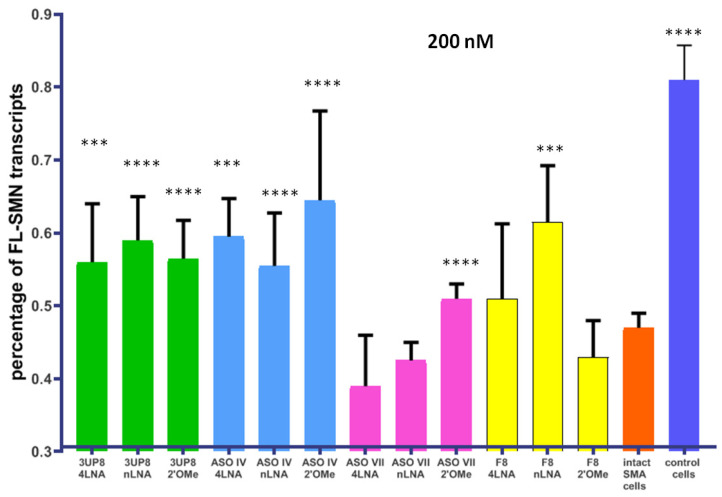
Percentage of full-length *SMN* transcripts in SMA fibroblast cell culture after delivery of AONs at 200 nM concentration. *** *p* < 0.001, **** *p* < 0.0001. Comparison was performed relative to intact cells. Medians with interquartile range are given.

We performed transfections of SMA fibroblast cells with ASO IV, and ASO VII at a concentration of 200 nM as well as positive control 3UP8 and negative control F8 modified by either 2′-O-methyl group or LNA (Figure 4). The presence of both 3UP8 and ASO IV in all variants led to a significant and robust enhancement in the levels of full-length *SMN* transcripts compared to the control cells. However, ASO VII with LNA did not demonstrate any positive impact. It is worth noting that the F8 AON, which we used as a negative control, actually resulted in an elevation of full-length *SMN* transcripts in the nLNA modification. None of the AONs have yet achieved the same level of *FL-SMN* transcripts as in fibroblast cell culture taken from a healthy individual.

**Figure 5:**
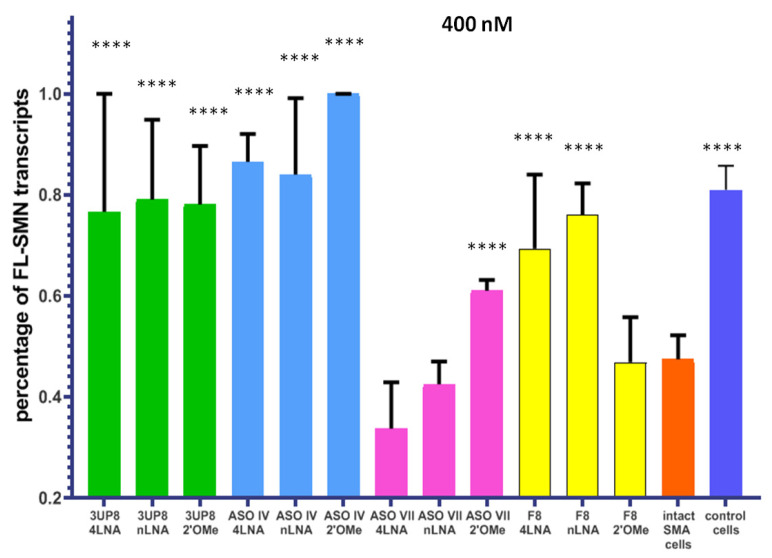
Percentage of full-length *SMN* transcripts in SMA fibroblast cell culture after delivery of AONs at concentration 400 nM. **** *p* < 0.0001. The comparison was performed relative to intact cells. Medians with interquartile range are given.

After the Alamar blue test showed no toxicity of these AONs to cells, we made the decision to increase the concentration of AONs to 400 nM (Figure S1a). Our goal was to investigate whether their efficiency would increase proportionally. Treatment of SMA cell cultures with the AONs, which had previously demonstrated a positive effect on splicing at a concentration of 200 nM, led to a remarkable increase in the levels of *FL-SMN* transcripts. On average, there was an improvement of 35% (ranging from 17% to 48%) (Figure 5). In addition, the 4LNA variant of F8 demonstrated a noteworthy impact, resulting in considerable enhancement of *FL-SMN* transcript levels. The SMA cell cultures that were transfected started producing *SMN* transcripts at a level comparable to that of healthy cells. No toxic effect on cells was detected at a 400 nM concentration as well (Figure S1b).

### 3.3. Simultaneous Targeting Two Nonadjacent SRE

**Figure 6:**
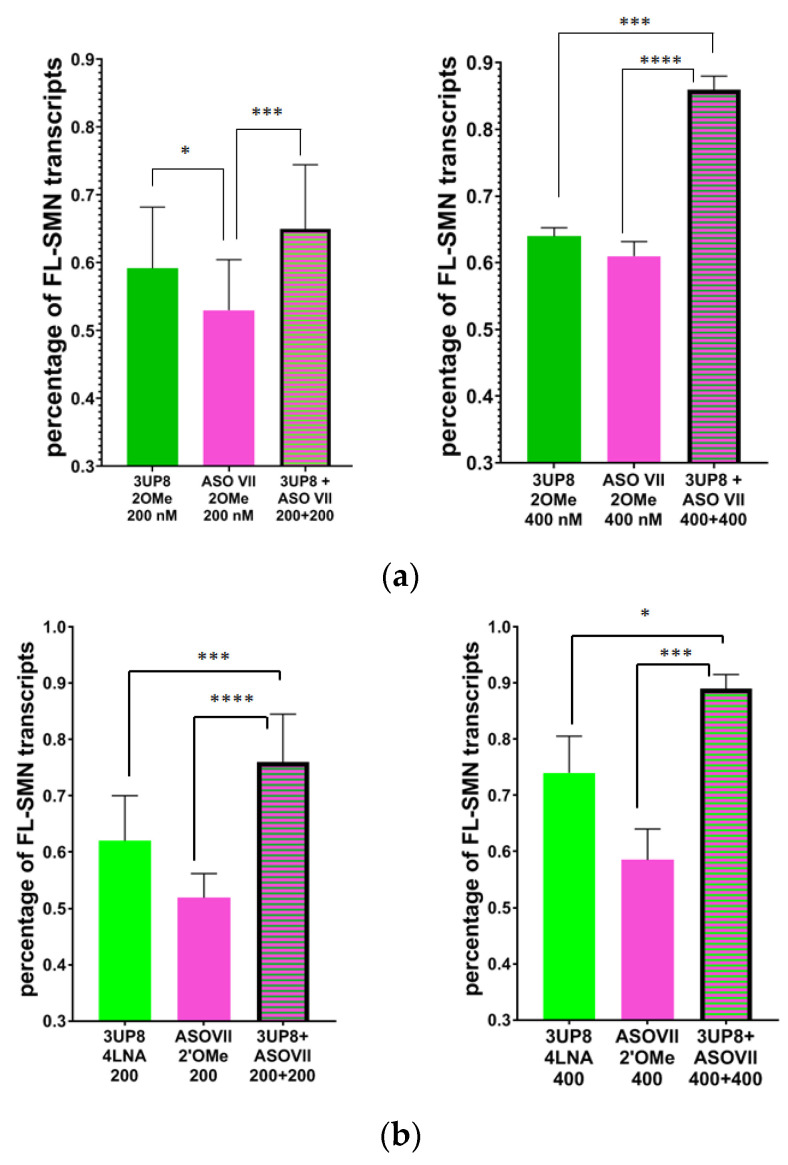

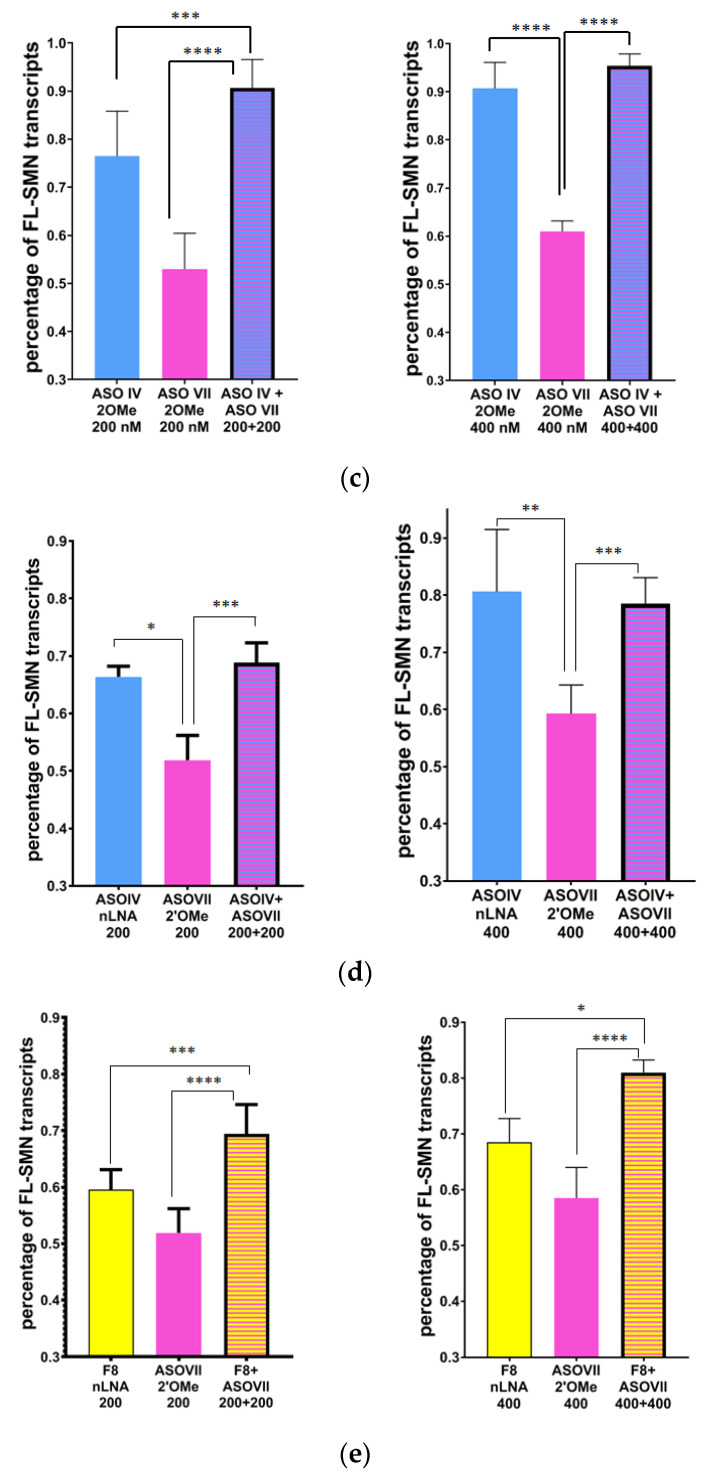
Percentage of full-length *SMN* transcripts in SMA fibroblast cell culture after delivery of AONs simultaneously vs. separately in 200 nM and 400 nM concentration. (**a**) 3UP8 2′OMe + ASO VII 2′OMe, (**b**) 3UP8 4LNA + ASO VII 2′OMe, (**c**) ASO IV 2′OMe + ASO VII 2′OMe, (**d**) ASO IV nLNA + ASO VII 2′OMe, (**e**) F8 nLNA + ASO VII 2′OMe. * *p* < 0.05, ** *p* < 0.01, *** *p* < 0.001, **** *p* < 0.0001. Medians with interquartile range are given.

We proceeded with transfecting cells using two distinct AONs that target nonadjacent splicing regulatory elements of *SMN2* exon 7. The purpose of this experiment is to evaluate the potential synergistic impact of splicing correction. We successfully utilized ASO VII 2′OMe, which effectively hinders an ISS in the A+100G region. In addition, we combined it with other AONs, namely 3UP8 2′OMe, 3UP8 4LNA, ASOIV 2′OMe, ASO IV nLNA, as well as F8 nLNA, that are complementary to ISS-N1. Since the effects of both 4LNA and nLNA modifications were similar, we decided to choose just one of these modifications for our upcoming experiments. We conducted tests on AONs at concentrations of 200 nM and 400 nM. The level of *FL-SMN* transcripts increased significantly in cells that were transfected with two AONs at the same time, specifically when using 3UP8 4LNA + ASO VII 2′OMe or F8 nLNA + ASO VII 2′OMe. This increase was much greater than in cells that received only one of these AONs, and the magnitude of the effect was dependent on the dose. This information is shown in Figure 6b,e. When both 3UP8 2′OMe and ASOVII 2′OMe were used together at a concentration of 400 nM, the results showed a significant and enhanced effect compared to delivering each of them individually (see Figure 6a). Simultaneous transfection of SMA cells with both ASO VII 2′OMe and ASO IV, modified with either 2′OMe or nLNA, proved to be more efficient compared to individual transfection. This increased efficiency was observed specifically in the 200 nM concentration. Nevertheless, when the concentration of ASO IV reached 400 nM, the abundance of *FL-SMN* transcripts was so remarkable that even the combined impact of the two AONs was unable to surpass it (Figure 6c,d).

### 3.4. Nuclear Gems Detection in Transfected SMA Fibroblasts

**Figure 7:**
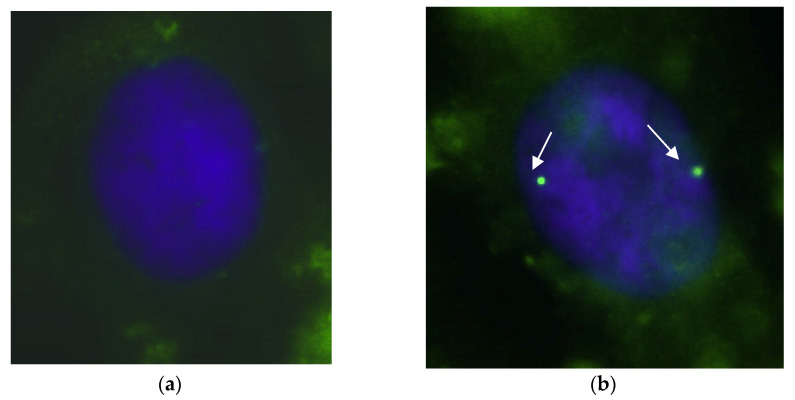
Visual appearance of gems in cell nucleus before (**a**) and after transfection with 3UP8 2′OMe 400 nM + ASO VII 2′OMe 400 nM (**b**) of SMA fibroblast cell cultures with AONs. Arrows indicate gems. The green color relates to SMN protein while the blue color relates to the Dapi-stained nucleus. Magnification ×1000.

**Figure 8:**
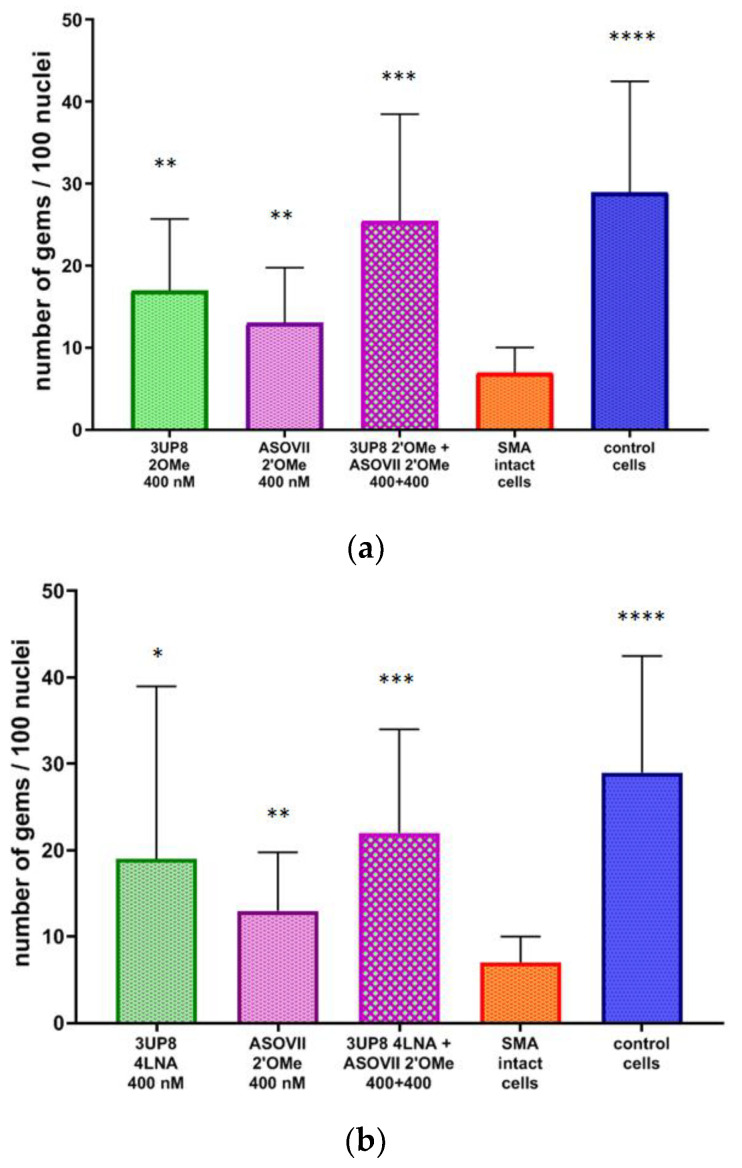

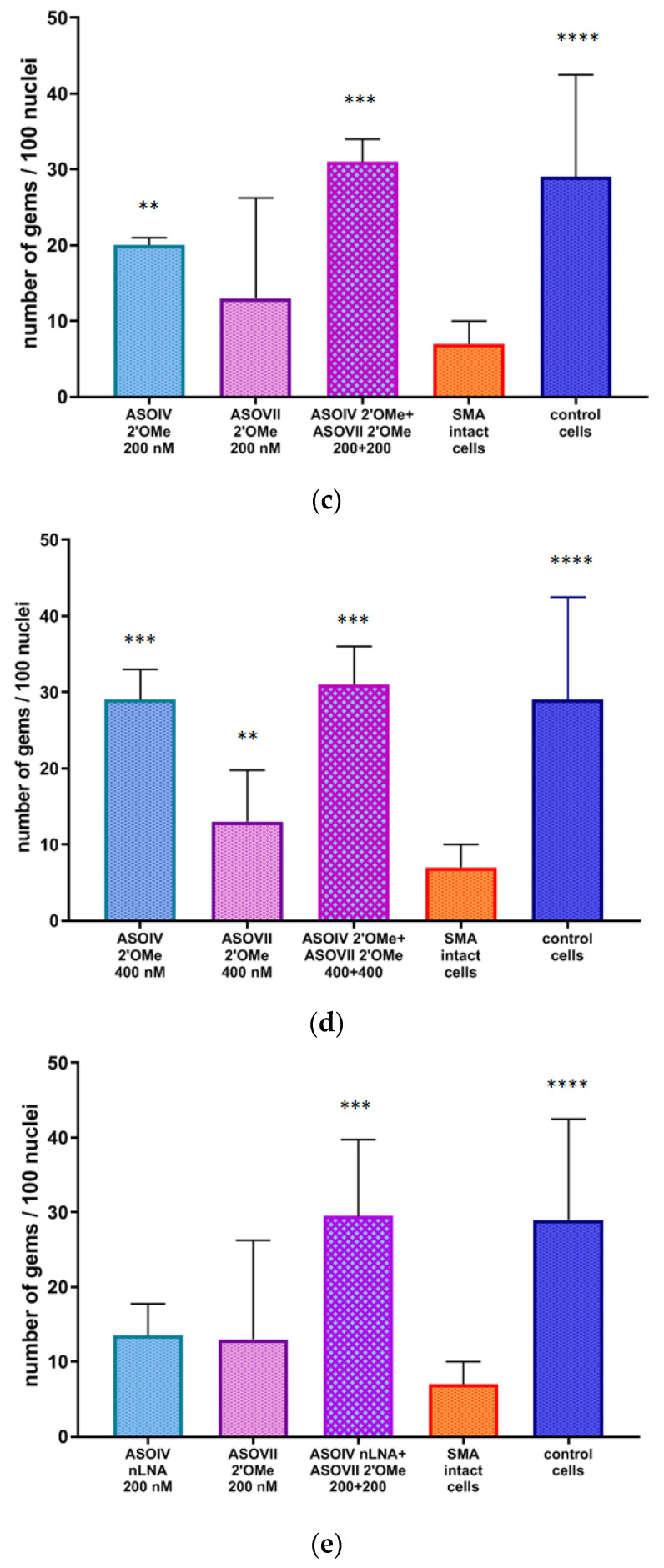

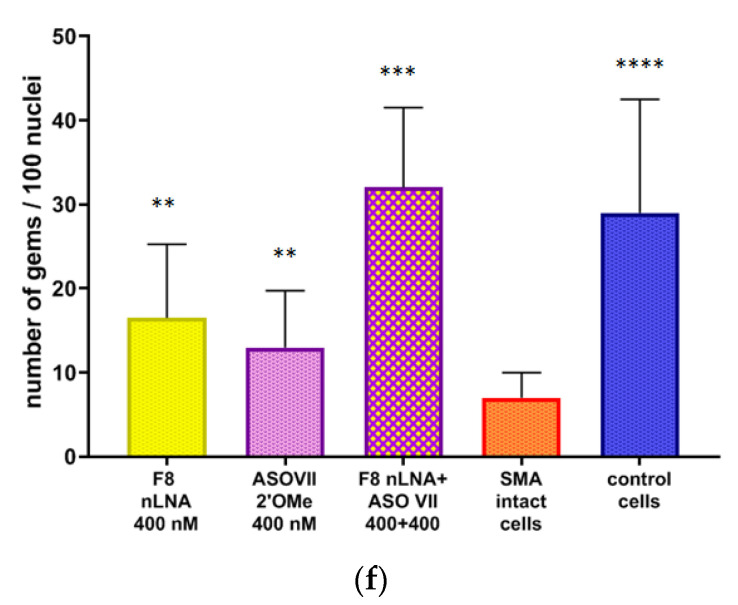
Number of gems per 100 nuclei in cultured SMA fibroblasts after treating with AONs vs. intact, as well as in cultured healthy fibroblasts. AONs used for the experiment: (**a**) 3UP8 2′OMe 400 nM + ASO VII 2′OMe 400 nM, (**b**) 3UP8 4LNA 400 nM + ASO VII 2′OMe 400 nM, (**c**) ASO IV 2′OMe 200 nM + ASO VII 2′OMe 200 nM, (**d**) ASO IV 2′OMe 400 nM + ASO VII 2′OMe 400 nM, (**e**) ASO IV nLNA 200 nM + ASO VII 2′OMe 200 nM, (**f**) F8 nLNA 400 nM + ASO VII 2′OMe 400 nM. * *p* < 0.05, ** *p* < 0.01, *** *p* < 0.001, **** *p* < 0.0001. Medians with interquartile range are given.

Additionally, we assessed the effects of the most effective pairs of AONs on the number of gems and observed a substantial increase in the amount of these structures that accumulate *SMN* in SMA cell cultures following transfection (Figure 7 and Figure 8).

Simultaneously targeting both ISS A+100G and ISS-N1 in most cases resulted in a significantly higher production of gems compared to the effect of downregulating only one of these sites (Figure 8a–c,e,f). The ASO IV 2′OMe oligonucleotide was the only exception to this, as it exhibited the same level of efficiency at a concentration of 400 nM, even without being combined with ASO VII 2′OMe (Figure 8d). This observation correlated with the results obtained in the analysis of *FL-SMN* transcripts level.

## 4. Discussion

In the current study, we designed and tested AONs complementary to elements regulating exon 7 splicing located in *SMN2* introns 6 and 7. For this study, certain AONs (ASOs II, III, VI, VII) were entirely original, while others had previously described sequences that were modified in a unique manner. In all the oligonucleotides a non-binding oxygen within the phosphate backbone was replaced with a sulfur thus forming phosphorothioate linkages. Such cost-effective modification demonstrates significant resistance to nuclease degradation [40]. The addition of 2′-O-methyl groups to a phosphorothioate-modified AON was shown to increase the stability of binding and reduce non-specific effects [41]. PS 2′OMe AON was widely studied in exon-skipping therapy research, demonstrating safety, efficacy, and specificity even higher than PMOs [42]. At the same time, PS 2′OMe monomers are easier to synthesize and cheaper compared to PMOs that are not compatible with standard phosphoramidite chemistry [43].

Another type of AON modification we tested was LNA which is also compatible with the standard solid-phase oligonucleotide synthesis procedures. LNA-modified oligonucleotides are characterized by high binding affinity to complementary sequences and increased stability in human serum thus improving the potency of AON for therapeutic application in vitro and in vivo [44,45]. The introduction of LNA nucleotides was demonstrated to allow shortening the length of AON thus helping to reduce the cost and potential toxicities of antisense-based therapy [43].

We have developed two schemes for PS LNA-containing AONs using a previously published algorithm [34,46]. The first scheme involves flanking the AONs with two LNA monomers on the 5’ and 3’ ends. The second scheme involves a pattern in which regular RNA nucleotides are interspersed with LNA nucleotides. According to the report by Shimo et al., the activity of splicing-regulating oligonucleotides was found to increase as more LNAs were added [47]. Additionally, this increase in activity was found to be correlated with the thermostability of AON. Moreover, the research unveiled that incorporating over 50% LNA monomers did not contribute to any significant increase in AON activity [47]. In PS 2′OMe oligonucleotides, every monomer was modified in the same way as described in previously conducted studies on SMA cell models [22].

When analyzing the effect of chosen PS LNA and PS 2′OMe oligonucleotides on *SMN2* splicing correction, we found that their impact on *FL-SMN* transcripts level rose with the increment of concentration from 200 nM to 400 nM. An interesting observation arose during the analysis of the effectiveness of oligonucleotides, which had the same sequence but varying chemical modifications. The positive impact of ASO VII on *SMN2* splicing vanished when LNA monomers were added; in contrast, the introduction of LNA nucleotides in the case of F8 AON triggered the splicing correction effect. The observation suggests a possible connection between the length of AONs containing LNA and their efficiency. Shorter AONs with LNA appear to demonstrate a greater level of efficacy compared to longer ones. Therefore, the smaller 8-mer F8 displayed enhanced effectiveness when modified with LNA, whereas the larger 20-mer ASO VII only proved to be efficient when modified with PS 2′OMe. The efficiency of splicing correction was slightly lower when introducing LNA monomers compared to the 2′OMe variant for the 18-nt-long ASO IV. The only AON that was similarly effective in all modifications was 3UP8.

Despite serving as a negative control when being PS 2′OMe modified, F8 is complementary to the hnRNP A1 binding site within ISS-N1 so the effect of this AON in splicing correction may be related to interaction with this region [22]. Adding LNA nucleotides in F8 might stabilize this molecule as well as interact with its target sequence. The findings emphasize the significance of every detail in the design of AONs. This includes the sequence composition, length, and type of chemical modification used.

In this study, we also demonstrated that targeting two non-adjacent splicing silencers simultaneously increases the dosage of full-length *SMN* transcripts and protein levels. It was shown for most AONs complementary to ISS-N1 and A+100G sites in *SMN2* pre-mRNA except ASO IV whose efficacy seemed to reach maximum in 400 nM concentration so that no cumulative effect with other AONs could overcome it.

The results of transcript analysis generally correlated strongly with the number of gems, which accurately reflects the level of SMN protein in the cell nuclei. Furthermore, it was previously demonstrated that the number of gems increases when SMA cells are treated with therapeutic molecules [15]. The reason behind the lack of noticeable variations in the number of gems in SMA fibroblasts treated with certain AONs (ASO IV nLNA and ASO VII 2′OMe at a concentration of 200 nM) compared to unaffected SMA fibroblasts can be attributed to the broader range of values for this biomarker when compared to transcripts. Additionally, the fewer repetitions involved in the immunocytochemical procedure may also contribute to this outcome. Nevertheless, we observed an increasing trend in the number of gems after AON-mediated splicing correction in all tested variants.

## 5. Conclusions

In our study, we have successfully shown how treating SMA-cultured fibroblasts with AONs that target splicing silencers in the 7 intron can lead to an increase in *FL-SMN* transcript levels and gems numbers. The main goal of SMA therapy is to boost the production of *SMN* on both the transcript and protein levels. Our research demonstrates that by selecting the right therapeutic molecule, we can elevate these molecular biomarkers to levels comparable to those found in healthy cells.

## Figures and Tables

**Table 1:** RNA AONs tested in the current study.

Name	Length, nt	Sequence	Reference
Phosphorothioate RNA AONs modified at each nucleotide by 2′-O-methyl moiety			
ASO I	20	5′-CUAUAUAUAGUUAUUCAACA-3′	[38]
ASO II	20	5′-CUAUAUAGAUAUAGAUAGCU-3′	this study
ASO III	20	5′-AAUAGCUAUAGAUAGCUUUA-3′	this study
ASO IV	18	5′-UCACUUUCAUAAUGCUGG-3′	[23]
ASO V	19	5′-ACCUUUCAACUUUCUAACA-3′	[30]
ASO VI	14	5′- UCAACUUUCUAACA-3′	this study
ASO VII	20	5′-UUCAACUUUCUAACAUCUGA-3′	this study
3UP8	8	5′-GCUGGCAG-3′	[22]
F8	8	5′-AAUGCUGG-3′	[22]
Phosphorothioate RNA AONs modified by LNA (indicated as capital letters)			
ASO IV 4LNA	18	5′-UCacuuucauaaugcUGg-3′	this study
ASO IV nLNA	18	5′-UcAcUuUcAuAaUgCuGg-3′	this study
ASO VII 4LNA	20	5′-UUcaacuuucuaacaucUGa-3′	this study
ASO VII nLNA	20	5′-UuCaAcUuUcUaAcAuCuGa-3′	this study
3UP8 4LNA	8	5′-GCuggCAg-3′	this study
3UP8 nLNA	8	5′-GcUgGcAg-3′	this study
F8 4LNA	8	5′-AAugcUGg-3′	this study
F8 nLNA	8	5′-AaUgCuGg-3′	this study

## Data Availability

The data are not publicly available due to restrictions of the subjects’ agreement.

## Supplementary Materials

The following supporting information can be downloaded at: https://www.mdpi.com/article/10.3390/biomedicines11113071/s1, Figure S1: The relative number of live fibroblasts (in percent) obtained from patients with SMA after transfection of AONs with various modifications at a concentration of 200 nM and 400 nM.
