# Supplementary material for: Development of 2′-O-Methyl and LNA Antisense Oligonucleotides for SMN2 Splicing Correction in SMA Cells
Source: Biomedicines. 2023 Nov 16;11(11):3071. doi: 10.3390/biomedicines11113071 (PMC10669464; doi:10.3390/biomedicines11113071)
Supplement: Supplementary file 1 [file biomedicines-11-03071-s001.zip › biomedicines-2665145-supplementary.pdf]

Supporting Information for the Article

Development of 2′-O-methyl and LNA antisense oligonucleotides for *SMN2* splicing correction in SMA cells

Marianna Maretina [1], Arina Il'ina [2], Anna Egorova [1], Andrey Glotov [1] and Anton Kiselev [1,*]

[1] Department of Genomic Medicine named after V.S. Baranov, D.O. Ott Research Institute of Obstetrics, Gynecology and Reproductology, Mendeleevskaya Line 3, 199034 Saint-Petersburg, Russia; M.M. marianna0204@gmail.com; A.E. egorova_anna@yahoo.com; A.G. anglotov@mail.ru

[2] Faculty of Biology, Saint-Petersburg State University, Universitetskaya Embankment 7-9, 199034, Saint-Petersburg, Russia; A.I. arina-ilina-23@yandex.ru

* Correspondence: ankiselev@yahoo.co.uk

Contents

Figure S1. The relative number of live fibroblasts (in percent) obtained from patients with SMA after transfection of AONs with various modifications at a concentration of 200 nM and 400 nM .....……………………………………………………………....3

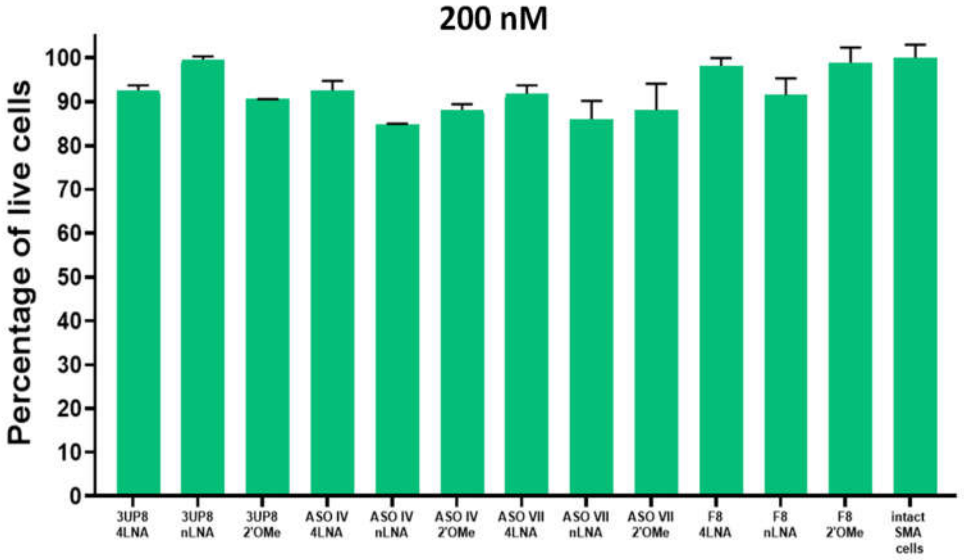

(a)

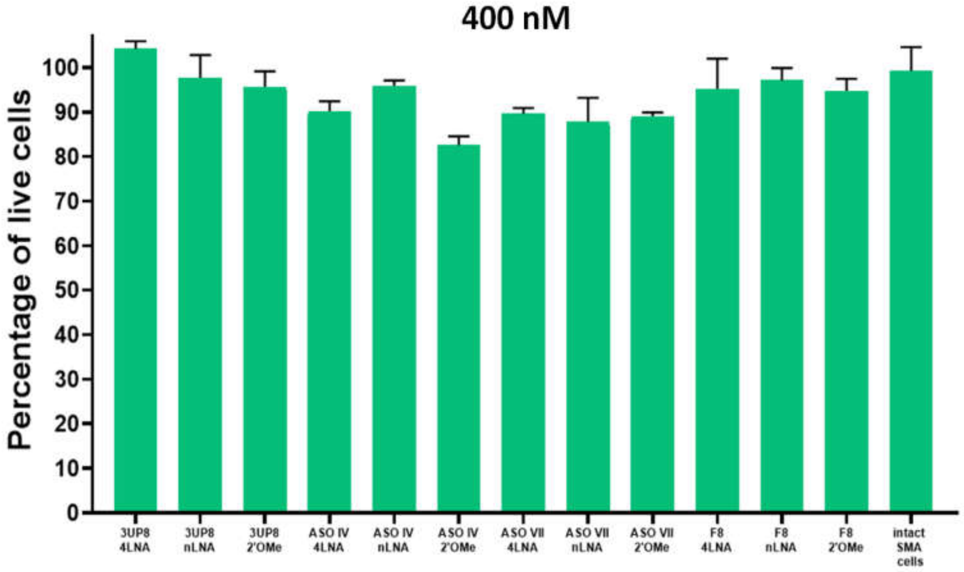

(b)

Figure S1. The relative number of live fibroblasts (in percent) obtained from patients with SMA after transfection of AONs with various modifications at a concentration of 200 nM (**a**) and 400 nM (**b**). The data were obtained using the Alamar Blue reagent. Medians with interquartile range are given.
